# Colon dysregulation in methamphetamine self-administering HIV-1 transgenic rats

**DOI:** 10.1371/journal.pone.0190078

**Published:** 2018-01-02

**Authors:** Amanda L. Persons, Brinda D. Bradaric, Hemraj B. Dodiya, Michael Ohene-Nyako, Christopher B. Forsyth, Ali Keshavarzian, Maliha Shaikh, T. Celeste Napier

**Affiliations:** 1 Department of Psychiatry, Rush University Medical Center, Chicago, IL, United States of America; 2 Department of Physician Assistant Studies, Rush University Medical Center, Chicago, IL, United States of America; 3 Center for Compulsive Behavior and Addiction, Rush University Medical Center, Chicago, IL, United States of America; 4 Department of Health Sciences, Rush University Medical Center, Chicago, IL, United States of America; 5 Department of Pharmacology, Rush University Medical Center, Chicago, IL, United States of America; 6 Department of Internal Medicine, Division of Digestive Diseases and Nutrition, Rush University Medical Center, Chicago, IL, United States of America; University of Nebraska Medical Center, UNITED STATES

## Abstract

The integrity and function of the gut is impaired in HIV-infected individuals, and gut pathogenesis may play a role in several HIV-associated disorders. Methamphetamine is a popular illicit drug abused by HIV-infected individuals. However, the effect of methamphetamine on the gut and its potential to exacerbate HIV-associated gut pathology is not known. To shed light on this scenario, we evaluated colon barrier pathology in a rat model of the human comorbid condition. Intestinal barrier integrity and permeability were assessed in drug-naïve Fischer 344 HIV-1 transgenic (Tg) and non-Tg rats, and in Tg and non-Tg rats instrumented with jugular cannulae trained to self-administer methamphetamine or serving as saline-yoked controls. Intestinal permeability was determined by measuring the urine content of orally gavaged sugars. Intestinal barrier integrity was evaluated by immunoblotting or immunofluorescence of colon claudin-1 and zonula occludens-1 (ZO-1), two major tight junction proteins that regulate gut epithelial paracellular permeability. Both non-Tg and Tg rats self-administered moderate amounts of methamphetamine. These amounts were sufficient to increase colon permeability, reduce protein level of claudin-1, and reduce claudin-1 and ZO-1 immunofluorescence in Tg rats relative to non-Tg rats. Methamphetamine decreased tight junction immunofluorescence in non-Tg rats, with a similar, but non-significant trend observed in Tg rats. However, the effect of methamphetamine on tight junction proteins was subthreshold to gut leakiness. These findings reveal that both HIV-1 proteins and methamphetamine alter colon barrier integrity, and indicate that the gut may be a pathogenic site for these insults.

## Introduction

Combined antiretroviral therapy (cART) is highly efficacious in controlling HIV plasma viral replication, and cART-adherence profoundly improves health. However, regardless of cART status, almost all HIV-infected individuals will eventually develop intestinal complications [1,2], a condition referred to as “HIV enteropathy” [3]. HIV enteropathy occurs during the acute phase of infection and throughout the advanced disease state, and it persists in patients on cART [1,4]. The enteropathy likely reflects infected lymphoblast cells evading the immune system that return to resting memory state during cART, but still harbor the HIV provirus and or viral DNA to form a viral reservoir in the gut [5,6]. A persistent viral reservoir participates in HIV pathogenesis [7], and impairment of the intestinal epithelial barrier may be involved. The structure of the intestinal epithelium is maintained by a complex interaction of tight junction proteins that regulate diffusion of toxins, microbes and various molecules from the lumen into the lamina propria and systemic circulation. Membrane-bound tight junction proteins (e.g., claudins and occludins) and their adapter and scaffolding protein, regulate paracellular diffusion, and claudins are the major determinants of paracellular transport processes [8]. Claudin-1 is largely localized in the apical region of colonic epithelial cells [9], and this transmembrane protein helps seal the epithelial monolayer. Zonula occuldens-1 (ZO-1) scaffolding proteins are localized in the cytoplasm and contain a binding domain that anchors claudin molecules to form a continuous paracellular seal. Reduction of ZO-1 expression disrupts tight junctions and results in barrier breakdown [10]. A reduction or morphological redistribution in sealing tight junction proteins can lead to intestinal hyperpermeability, so that contents normally restricted to the lumen (e.g., lipopolysaccharide, LPS) may translocate into the lamina propria and circulation to promote systemic inflammation [11]. Systemic inflammation may further dysregulate the gut, exacerbate gut pathology, and ultimately worsen disease. In HIV-infected patients, reduced tight junction proteins such as claudin-1, claudin-7 and ZO-1 in the colon and small intestine is associated with increased intestinal permeability, increased LPS translocation, and increased systemic inflammation [12].

Methamphetamine is a popular illicit drug, and its use by HIV-infected individuals is associated with a more rapid progression of HIV-related diseases [13]. The effect of methamphetamine on tight junction proteins in the gut is unknown. But methamphetamine is known to disrupt another critical barrier, the blood brain barrier, causing increased permeability [14–16] and impairing tight junction proteins [17–19]. Case reports link methamphetamine use to ischemic colitis [20–22] and vasculitis of the distal colon [23]. Such gut pathologies may reflect inflammation brought about by disruption of the intestinal barrier. Taken together, these reports indicate that methamphetamine may alter intestinal tight junction proteins and promote intestinal leakiness.

The interactions between methamphetamine abuse and HIV disease progression are complex and remain unclear [13]. However, the common intestinal symptomology observed in both HIV-infected patients and methamphetamine users supports our hypothesis that the gut is a site of comorbidity. To address this hypothesis, we studied the intestine from rats that model key aspects of the human comorbid condition, i.e., HIV-1 transgenic (Tg) rats trained to self-administer methamphetamine. Tg rats express 7 of the 9 HIV-1 proteins independent of viral replication and thus provide a useful means to model HIV-infected patients with cART-controlled viral replication [24]. To model humans abusing methamphetamine, rats were allowed to self-administer the drug. To determine gut barrier function, the permeability of the colon was determined by assessing orally gavaged sugar probes excreted in the urine. Colon barrier integrity was also assessed by immunoblotting or staining for claudin-1 and ZO-1 protein in the colon epithelium. Study outcomes reveal changes in colon barrier function and integrity within the colon of HIV-1 Tg rats and demonstrate colon hyper-permeability in the comorbid condition.

## Materials and methods

### Animals

Twenty male HIV-1 Tg Fischer 344 rats (Envigo Laboratories, Indianapolis, IN) were purchased at 3–4 weeks of age and raised in the Rush University vivarium. Twenty male non-Tg Fischer 344 rats were age-matched to the Tg rats and purchased (Envigo Laboratories) two weeks prior to experimentation. All rats were housed in pairs in the environmentally-controlled (12h light/dark cycle; lights on at 7AM) with food and water available *ad libitum*. At the end of each experiment, rats were intraperitoneally (ip) administered chloral hydrate (400mg/kg). This dose of ip chloral hydrate produces sedation within minutes [25,26], responding to moderate aversive stimulation (e.g., toe pinch) is lost by 15min post injection, and deep anesthesia adequate for surgical procedures (e.g., loss of responding to skin incision [27], alterations in brain activity consistent with anesthesia [28]) occurs within 30min post-injection and persists for 90min post-injection [25]. Thus, at approximately 30min post injection and following complete loss of responding to a strong paw pinch and chest skin incision, the rats were killed *via* pneumothorax and transcardially perfused with saline. All procedures were performed in accordance with the *Guide for the Care and Use of Laboratory Animals* (National Research Council, Washington DC) and approved by the Rush University Institutional Animal Care and Use Committee.

### Self-administration procedure

#### Implantation of intravenous catheters

Procedures for catheter implants followed our previously published protocols [29–31]. In brief, rats (n = 40; weighting at least 250g) were anesthetized with 2–3% isoflurane and implanted with custom built catheters constructed of silastic tubing (0.3mm ID × 0.64mm OD; Dow Corning Co., Midland, MI) in the right jugular vein. The opposite end of the cannula was passed subcutaneously over the mid-scapular region and exited through a metal guide cannula (22 gauge; Plastics One Inc., Roanoke, VA). Rats were allowed to recover from surgery for 10–14 days. Topical antibiotic (bacitracin) was applied as needed. Catheters flushed daily with 0.1–0.2ml sterile saline to maintain patency, implied by ease of flushing and consistent methamphetamine self-administration.

#### Self-administration

Operant chambers (Med-Associates, St. Albans, VT) were equipped with two ‘nose-poke’ holes, a stimulus light above each hole, an audio tone generator, and a house light. Each operant chamber was enclosed in a ventilated, sound-attenuating chamber. Infusions were delivered *via* syringe in a motor-driven pump. Self-administration sessions were conducted 2h/day for a total of 21 days.

A nose-poke in the ‘active’ hole (left hole) resulted in a 6s infusion of (+)methamphetamine HCl (0.02mg/kg per 0.05mL sterile saline; Sigma-Aldrich, St. Louis, MO) on a fixed-ratio 1 (FR1) schedule of reinforcement. During the infusion, the cue light above the active hole was illuminated and a 65dB tone was generated. During this time, additional nose-pokes were recorded but were not reinforced. There was no post-infusion timeout period. For self-administration days 8–21, the infusion of methamphetamine was increased to 0.04mg/kg per 0.05mL sterile saline and remained on a FR1 schedule of reinforcement. Nose-pokes in the ‘inactive’ hole (right hole) had no programmed consequence. Control rats were saline-yoked to a methamphetamine counterpart of the same genotype; these rats received a non-contingent infusion of saline (0.05mL) accompanied by a light and tone cues each time their methamphetamine counterpart received a methamphetamine infusion. For saline-yoked rats, nose-pokes in either hole were recorded but had no programmed consequence. One Tg rat died from undetermined causes during the self-administration paradigm.

### Intestinal permeability

Intestinal permeability was assessed as previously published [32,33] in naïve (i.e., un-instrumented) rats (12 weeks of age), and rats that underwent self-administration procedures (17–22 weeks of age). For the latter, permeability testing was conducted one day after the last operant session. Briefly, all rats were fasted overnight prior to permeability testing. After the fast, rats were given a 1.5mL sugar cocktail containing sucrose (142.5mg), mannitol (7.5mg), lactulose (26.75mg) and sucralose (3.75mg) *via* oral gavage. To promote urine output, each rat received 8mL of lactated Ringer’s solution subcutaneously, immediately following oral sugar administration. Rats were housed individually in metabolic cages, and urine was collected for 5h.

Urine sugar levels were measured by gas chromatography as previously described [34,35]. Briefly, urine samples (100μL) were mixed with 1mg myo-inositol as the internal standard. The standard tube contained 1mg of the 4 sugar cocktail (sucrose, lactulose, mannitol, and sucralose). All samples were hydrolyzed with 2M trifluoracetic acid; hydrolyzed samples were then reduced by dissolving in 1M ammonium hydroxide. A solution of dimethyl sulfoxide containing 20mg/mL of sodium borodeuteride was added to the samples and kept at 40°C for 90min. For O-acetylation, 100μl of 1-methylimidazole and 0.5mL of acetic anhydride was added to the samples followed by a mixture of 4mL water/1mL methylene chloride. The samples were dried and the final residue dissolved in 0.5mL acetone for gas chromatography. Gas chromatography was performed using an Agilent 6890 GC equipped with a flame ionization detector with a DB-225MS column (30m × 250μm ID with a 0.25 film thickness). The detector and injector temperatures were set at 300°C and 240°C respectively. The initial column temperature of 100°C was held for 2min and then increased at a rate of 10°C/min and held at 180°C for another 2min. The column temperature was further increased at a rate of 4°C /min to 240°C, which was maintained for 15min. The total run time was 42min.

### Immunoblotting

Colon tissues (20mg) were washed in PBS and centrifuged; the supernatant was discarded. Membrane and cytoplasmic fractions were extracted from the pellets using the NE-PER^TM^ kit (ThermoFisher Scientific, Waltham, MA). The supernatant (cytoplasmic extract) was removed and the pellet (containing the membrane fragments) suspended in 100μl of tris-triton buffer. Samples were incubated on ice for 20min and then centrifuged (16000xg for 10min). The supernatant was collected and stored at -80°C. Protein concentration was determined using the Bradford assay [36].

Homogenized colon samples (10μg) were boiled at 100°C for 5mins with 2x Laemmli sample buffer (Bio-Rad Laboratories, Hercules, CA). Samples were electrophoresed on 15% tris-HCl gels and transferred to 0.2μm nitrocellulose membranes. Non-specific binding sites were blocked using 2.5% bovine serum albumin and 2.5% non-fat dry milk in tris-buffered saline ⁄ Tween-20 (TBS-T), for 1h at room temperature (RT). Membranes were incubated overnight at 4°C with claudin-1 primary antibody (1:1000, Santa Cruz Biotechnology, Dallas, TX) in TBST. Membranes were incubated in HRP-conjugated rabbit anti-mouse secondary antibody (1:2000, Jackson ImmunoResearch, West Grove, PA) for 1h at RT. Protein bands were visualized using Pico chemiluminescent substrate (ThermoFisher Scientific). Membranes were stained with a Ponceau S solution (Sigma-Aldrich, St. Louis, MO) and used as the loading control. Films and Ponceau S-stained membranes were scanned and optical density determined using ImageJ software (NIH, Bethesda, MD).

### Immunofluorescence

Distal colons were excised from all rats, extensively flushed with phosphate-buffered saline, embedded into optimal cutting temperature compound, and flash frozen. Embedded colon samples were stored at -80°C. Two distal colon sections (20μm) were evaluated for each rat. Sections were post-fixed with acetone (-20°C for 15min), air-dried, and incubated in 3% serum (specific serum targeting host of the secondary antibody) and 2% bovine serum albumin for 1h before overnight incubation with primary antibody for claudin-1 (1:50) or ZO-1 (1:100) for 24h at room temperature. Sections were washed and incubated with rhodamine-conjugated secondary antibody (1:100) or Alexa Fluor® 555 (1:400) for 1h at room temperature. All sections were counter-stained with DAPI (5min, 1:10,000) to identify cell nuclei. All antibodies were sourced from ThermoFisher Scientific.

### Quantification and statistical analyses

The number of active nose-pokes, inactive nose-pokes and infusions were averaged across all sessions. The active and inactive nose-pokes were analyzed using a two-way ANOVA with Newman-Keuls *post-hoc* comparisons. The average number of infusions per session and cumulative methamphetamine intake were analyzed using a non-directional Student’s *t*-test.

The urine sucralose/lactulose ratio and claudin-1 protein expression (immunoblots) were analyzed using a two-way ANOVA with genotype and treatment as factors. A *post hoc* Newman-Keuls test was used for between-group differences; planned contrasts selected for comparisons were treatment within each genotype, and treatment between genotypes. Claudin-1 and ZO-1 immunofluorescence were scored by two genotype and/or treatment-blinded observers using images magnified 20x on a Zeiss confocal or an Olympus microscope. A rating scale was developed that accounted for intensity and distribution of tight junction immunofluorescence as follows: 0 = no immunofluorescence, 1 = light, discontinuous immunofluorescence, 2 = light, continuous immunofluorescence, 3 = intense, discontinuous immunofluorescence, 4 = intense, continuous immunofluorescence around the crypts. Within each section, 15–20 crypts were identified, and immunofluorescence was scored. Scores were analyzed using a corrected one-tailed, non-parametric Mann-Whitney *U*-test (α = 0.025) to allow for the multiple planned contrasts (listed above). All data were analyzed using GraphPad Prism 5.0 (GraphPad Software, La Jolla, CA) or GB-Stat (Dynamic Microsystems, Silver Spring, MD) and are represented as mean+SEM.

## Results

### Methamphetamine self-administration

**Fig 1:**
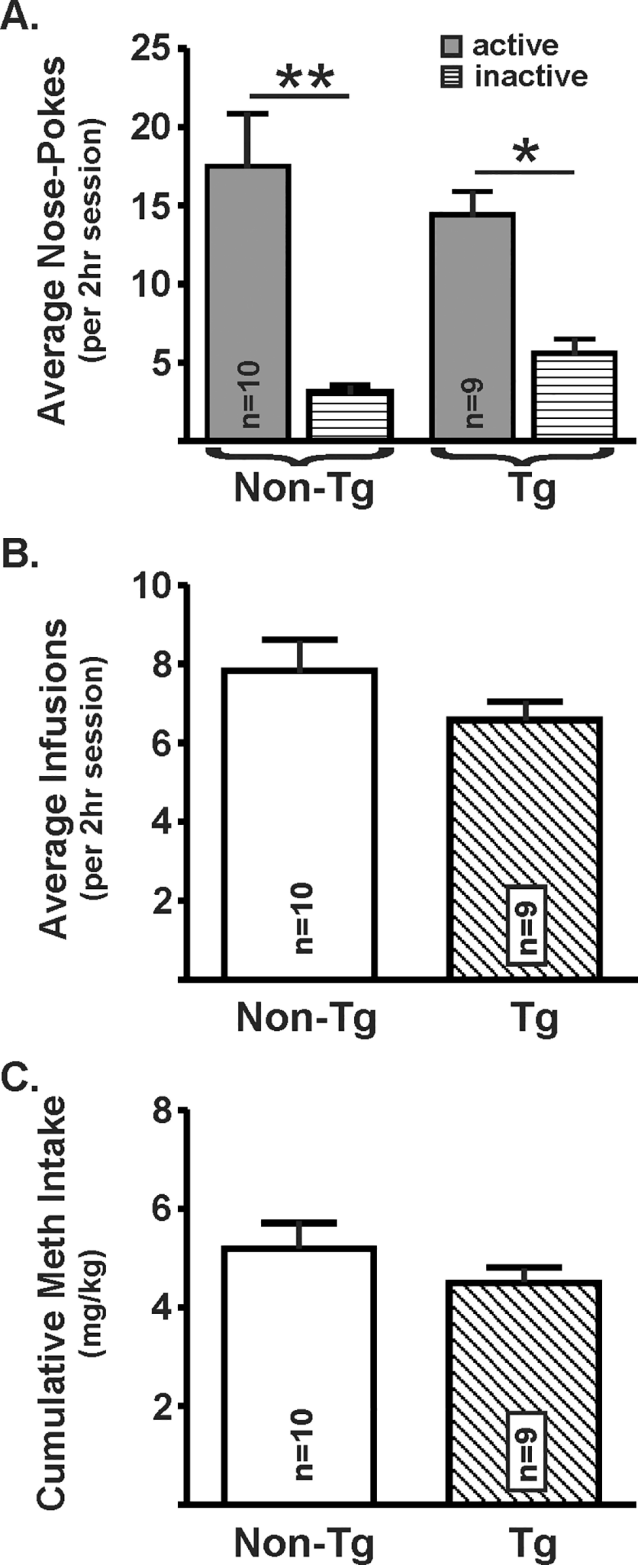
Methamphetamine self-administration in non-Tg and Tg rats. **(A)** Non-Tg and Tg rats similarly performed the methamphetamine self-administration task. Shown is the average number of nose-pokes per 2h session across 21 days by non-Tg and Tg rats. There were a greater number of active nose-pokes *vs* inactive nose-pokes for both genotypes. Non-Tg and Tg rats did not differ in the **(B)** average number of methamphetamine infusions per session, or **(C)** total methamphetamine intake (Student’s *t*-test, * *p*<0.05; ** *p*<0.01).

Non-Tg and Tg rats similarly acquired and maintained the operant task necessary for self-administration of methamphetamine. There was a significant increase in the number of nose-pokes in the active hole compared to the inactive hole (F_(1,17)_ = 35.9, *p*<0.0001), independent of genotype (F_(1,17)_ = 0.03, *p* = 0.88; Fig 1A). There also was no difference between genotypes in the average number of infusions per session (t_(17)_ = 1.38, *p* = 0.2; Fig 1B) or total cumulative methamphetamine intake (t_(17)_ = 1.11, *p* = 0.28; Fig 1C). Additionally, we determined that there was no effect of genotype on the number of methamphetamine infusions across the sessions (genotype: F_(1,17)_ = 1.82, *p* = 0.19; time: F_(20,340)_ = 11.65, *p*<0.0001; interaction: F_(20,340)_ = 2.98, *p* = 0.76). Saline-yoked rats received equal numbers of saline infusions as their methamphetamine self-administering counterparts.

### Effect of genotype and methamphetamine self-administration on intestinal permeability

**Fig 2:**
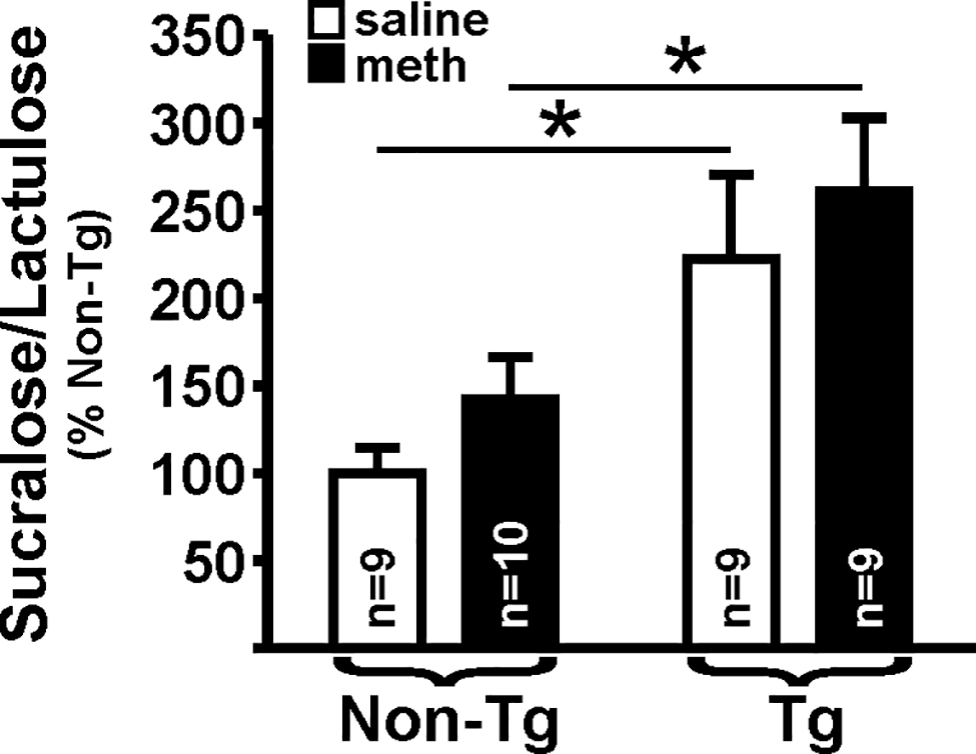
Colon permeability. Tg rats, regardless of methamphetamine self-administration, exhibited a significantly more permeable colon, as assessed the by the sucralose/lactulose ratio, compared to non-Tg saline yoked-rats (*p* = 0.001). Methamphetamine self-administration did not result in a significant difference (*p* = 0.25), and there was no interaction between genotype and meth (*p* = 0.96). Horizontal lines indicate planned contrasts that showed differences with a Newman-Keuls *post hoc* test (* *p*<0.05).

Intestinal permeability was assessed in non-Tg and Tg rats that self-administered methamphetamine and saline-yoked controls. For the functional permeability assessment (Fig 2), there was a significant effect of genotype (F_(1,33)_ = 12.38, *p* = 0.001) on the sucralose/lactulose ratio, which confirmed our preliminary findings [37]. However, there was no effect of treatment (F_(1,33)_ = 1.4, *p* = 0.25) and no interaction (F_(1,33)_ = 0.003, *p* = 0.96). *Post hoc* analysis indicated a significant difference between non-Tg and Tg rats, for both saline-yoked and methamphetamine treatment groups.

### Effect of genotype and methamphetamine self-administration on barrier integrity

**Fig 3:**
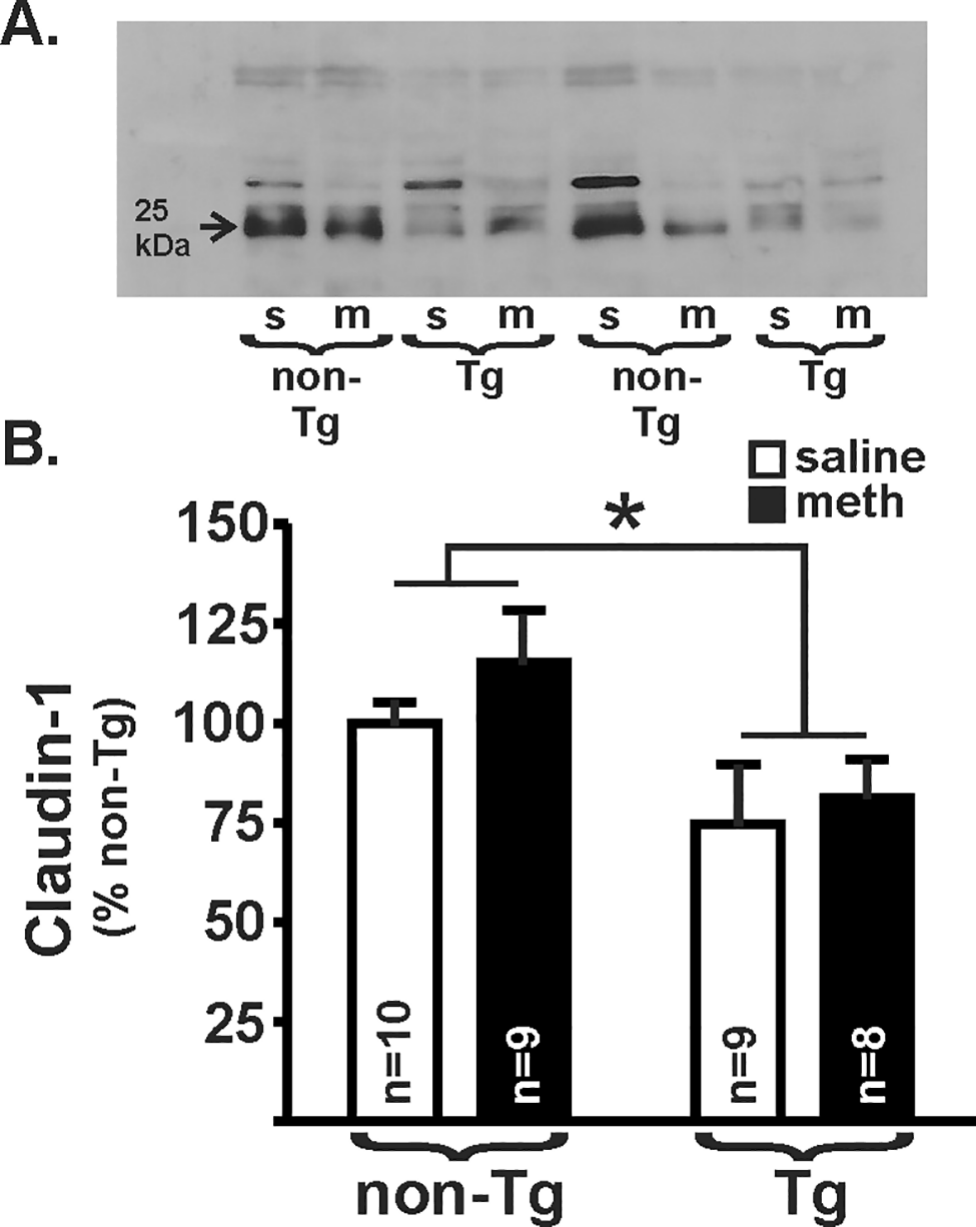
Claudin-1 protein expression. **(A) I**mmunoblot illustrating claudin-1 (23kDa) in the distal colon from 8 different samples (s, saline; m, meth). Arrow indicates molecular weight at 25kDa. **(B)** Overall, Tg rats had a significantly less claudin-1 compared to non-Tg rats (*p* = 0.01). Methamphetamine (meth) self-administration did not result in a significant difference (*p* = 0.34), and there was no interaction between genotype and meth (*p* = 0.70). Horizontal lines indicate an overall effect of genotype from the two-way ANOVA (* *p*<0.05).

**Fig 4:**
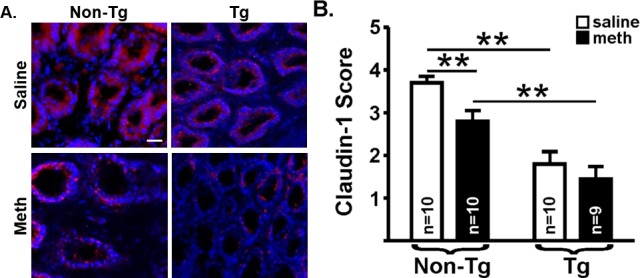
Claudin-1 immunofluorescence. **(A)** Representative photomicrographs of claudin-1 immunofluorescence (red) in the distal colon of saline-yoked **(top)** and methamphetamine (meth) self-administering **(bottom)** non-Tg **(left)** and Tg **(right)** rats. Sections were counterstained with DAPI (blue) to identify crypts. Colon tissue from saline-yoked non-Tg rats exhibited continuous, intense claudin-1 immunofluorescence around the epithelial cells within the crypts **(top, left)**; whereas colons from saline-yoked Tg rats exhibited light, continuous claudin-1 immunofluorescence (**top, right**). Non-Tg rats that self-administered methamphetamine had discontinuous claudin-1 immunofluorescence (**bottom, left**). Tg rats that self-administered methamphetamine (**bottom, right**) displayed the least amount of claudin-1 immunofluorescence. White bar = 25μm. **(B)** A scale from 0–4 was used to score claudin-1 immunofluorescence (see Methods). As indicated by horizontal lines above the bars, scores differed significantly between saline-yoked (open bars) non-Tg and saline-yoked Tg rats, methamphetamine self-administering (filled bars) non-Tg and Tg rats, and saline-yoked non-Tg and methamphetamine self-administering non-Tg rats. Mann-Whitney *U*-tests corrected for multiple comparisons (** *p*<0.01).

**Fig 5:**
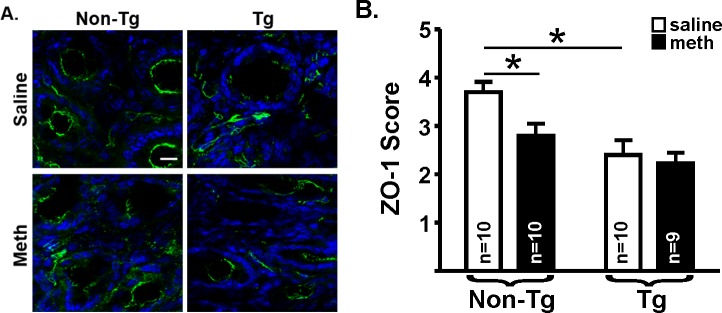
ZO-1 immunofluorescence. **(A)** Representative photomicrographs of ZO-1 immunofluorescence (red) in the distal colon of saline-yoked **(top)** and methamphetamine self-administering **(bottom)** non-Tg **(left)** and Tg **(right)** rats. Sections were counterstained with DAPI (blue) to identify crypts. Saline-yoked non-Tg exhibited continuous ZO-1 immunofluorescence around the epithelial cells within the crypts **(top, left)**; whereas non-Tg rats that self-administered methamphetamine had discontinuous ZO-1 immunofluorescence **(bottom, left).** Saline-yoked non-Tg and Tg rats that self-administered methamphetamine also had discontinuous ZO-1 immunofluorescence **(right)**. White bar = 25μm. There was no group difference in intensity of ZO-1 immunofluorescence. **(B)** A scale from 0–4 was used to score ZO-1 immunofluorescence (see Methods). As indicated by horizontal lines above the bars, scores differed significantly between saline-yoked (open bars) non-Tg and saline-yoked Tg rats, and saline-yoked non-Tg and methamphetamine self-administering (filled bars) non-Tg rats. Mann-Whitney *U*-tests corrected for multiple comparisons (* *p*<0.025).

Our pilot suggested that increased colon permeability may be due to a loss of barrier integrity that is maintained by tight junction proteins [37]. Thus, here we used membrane fractions of distal colon homogenates to assess levels of claudin-1 using immunoblots (Fig 3). There was a significant effect of genotype on claudin-1 expression (F_(1,32)_ = 6.87, *p* = 0.01), but no effect of treatment (F_(1,32)_ = 0.91, *p* = 0.34) and no interaction (F_(1,33)_ = 0.15, *p* = 0.70), similar to functional permeability outcomes. To determine if colon leakiness was due to an overall loss of tight junctions or a change in the distribution of tight junction protein expression, we used immunofluorescence to qualify the staining patterns of claudin-1, as well as a second tight junction protein, ZO-1. The integrity of the intestinal barrier in the non-Tg saline-yoked rats appeared to be intact, as indicated by the continuous and intense claudin-1 (Fig 4A) and ZO-1 (Fig 5A) immunofluorescence around the crypts. Relative to these non-Tg saline-yoked rats, saline-yoked Tg rats exhibited significantly altered claudin-1 (*U* = 3.0, *p* = 0.0001; Fig 4B) and ZO-1 (*U* = 20.0, *p* = 0.015; Fig 5B) immunofluorescence around the colonic crypts. Also compared to non-Tg saline-yoked rats, non-Tg rats that self-administered methamphetamine exhibited significantly altered claudin-1 (*U* = 19.0, *p* = 0.007) and ZO-1 (*U* = 13.5, *p* = 0.004) immunofluorescence, wherein the intensity of staining was normal, but the staining pattern was discontinuous. Methamphetamine self-administering Tg rats had light *and* discontinuous claudin-1 immunofluorescence (see Fig 4A, lower right), which was significantly different from methamphetamine self-administering non-Tg rats (*U* = 12.0, *p* = 0.003). In contrast, ZO-1 immunofluorescence in both the methamphetamine self-administering and saline-yoked Tg rats was intense, however discontinuous (see Fig 5A, lower right). There was no significant difference in claudin-1 immunofluorescence score (*U* = 34.0, *p* = 0.18) or ZO-1 (*U* = 28.0, *p* = 0.07) between the Tg saline-yoked or Tg methamphetamine self-administering rats.

## Discussion

Using the non-infectious HIV-1 Tg rat model of HIV-infected humans on cART, we describe increased colon barrier permeability associated with the expression of toxic HIV-1 proteins and alteration of tight junction protein expression. We reveal that HIV-1 Tg rats recapitulated the colon permeability seen in HIV-infected patients [38,39]. We also reveal that methamphetamine altered claudin-1 and ZO-1 expression in the colon. However, colon pathology in the comorbid scenario was not greater than that found with either condition alone, i.e., in HIV-1 Tg rats given saline or with methamphetamine exposed non-Tg rats.

Animal models that recapitulate key aspects of human disease are critical for understanding the biology of the disease, as well as for medication development. There are several rodent models of HIV-infection, and of methamphetamine addiction. Regarding the former, the humanized mouse model recapitulates important pathologies of HIV/AIDS such as depletion of CD4^+^ T cells, generalized immune activation [40], and gut barrier pathology [41]. However, these mice retain innate immune responses and exhibit difficulty in generating memory T cells [42], which may influence outcomes. Mice that express single genes, such as the HIV-1 proteins gp120 or Tat, allow evaluation of the effects imposed by only one of seven identified toxic HIV-1 proteins. HIV-1 Tg rats express all seven of the toxic viral proteins, the two genes responsible for infection and replication (*gag-pol*) are absent [43]. These rats recapitulate viral protein expression patterns seen in HIV-infected humans, especially those maintained on anti-retroviral regimens [24]; therefore, these rats are used to study HIV/AIDS-related pathologies [44]. Another factor that influenced the choice of model was the capacity of the rats to acquire and perform self-administration operant tasks (discussed below), which are more difficult for mice.

Study outcomes support the validity of HIV-1 Tg rats as a model for gut pathology seen in HIV-infected humans. We revealed, that similar to the clinical scenario [38,39], colon barrier function and integrity is impaired in HIV-1 Tg rats as compared to non-Tg rats. Recently, Banerjee *et al*. [45] reported gut leakiness, i.e., increased serum LPS in HIV-1 Tg rats exposed to binge alcohol treatment. In contrast to our results, alcohol-free Tg rats did not show changes in gut permeability compared to non-Tg un-treated rats. This discrepancy may be due to the fact that our rats were 17–22 weeks of age and males, whereas the rats used in the Banerjee study were 6–8 weeks old and females. As HIV-1 Tg rats show age-dependent differences in HIV-1 protein mRNA expression [24], it is possible that the duration of exposure to viral proteins may determine the extent of barrier damage, such that older rats will show enhanced barrier pathology relative to younger rats. Sex may also be a discriminating factor. Estrogen improves esophageal barrier function and reduces epithelial barrier permeability by potentiating occludin expression in rabbits [46]. Estrogen also attenuates oxidative stress induced by the HIV-1 proteins Tat and gp120 [47]. It may be that estrogen produced by the female Tg rats used by Banerjee *et al*. [45] may have conferred protection against the effect of the viral proteins on the colon epithelial barrier.

To assess functional colon status, we evaluated the translocation of small sugar probes. The use of small sugar probes to evaluate intestinal paracellular permeability is simple and non-invasive [48]. Sucralose is stable throughout the gastrointestinal tract, and lactulose evaluates small intestine permeability. Thus, the ratio of sucralose/lactulose specifically assesses colon barrier permeability [35]. HIV-1 Tg rats had a higher urine sucralose/lactulose ratio compared to non-Tg rats. This observation recapitulates the enhanced colon permeability seen in HIV-infected humans [38,39].

To indicate a mechanism that underlies gut leakiness imposed by chronic exposure to HIV-1 proteins, we evaluated protein expression using immunoblotting and immunofluorescence for the tight junction protein, claudin-1, in the colon of saline-yoked HIV-1 Tg and non-Tg rats. There was an overall decrease in claudin-1 protein in the distal colon of Tg rats; i.e., immunofluorescence demonstrated lighter intensity staining that was discontinuous in pattern in the Tg rats, suggesting abnormal expression and distribution of claudin-1. These observations support and extend a preliminary study by Zhang *et al*. [41], in which colon claudin-2, a pore-forming claudin (that regulates permeability), was increased in the humanized mouse model of HIV. The current findings also concur with studies of humans by Chung *et al*. [38] who reported a dysregulation of claudin-2 and -4, and Tincati *et al*. [39] who reported a reduction in claudin-1 and -7 in biopsied colonic tissues from HIV-infected patients on antiretroviral therapy, relative to healthy controls. We also evaluated ZO-1, an intracellular scaffolding protein that organizes and maintains tight junction proteins by linking transmembrane proteins, such as claudin-1, sealing paracellular transport [10]. In the colon of saline-yoked Tg and non-Tg rats, there was a decrease in ZO-1 staining. However, following methamphetamine exposure, the pattern and distribution of staining was discontinuous, regardless of genotype. In a recent study by Patel *et al*, exposure to Tat and methamphetamine altered ZO-1 expression in an *in vitro* model of the blood-brain barrier; however, alterations in functional permeability were not obtained [49]. This suggests that alterations in the distribution and expression of multiple tight junction proteins may play a role in the functional permeability that was observed in the current *in vivo* study with non-Tg and Tg rats.

To determine the effect of methamphetamine use during exposure to HIV-1 proteins on the outcomes of colon pathology, we utilized a contingency operant paradigm wherein HIV-1 Tg rats self-administered methamphetamine. To our knowledge, there are four published studies on methamphetamine in HIV-1 Tg rats [50–53]. In all four, non-contingent administration approaches were used, with doses that are significantly greater than the daily intake of methamphetamine self-selected by the rats. Allowing rats to self-titrate methamphetamine in the context of visual and audio cues emulates the motivational aspects of drug-taking in humans, and controls for the known contributions imposed by task contingencies that influence behavioral and biochemical readouts of drug exposure in rats [54,55]; for review, see [56]). Fischer 344 rats exhibit a low motivational state; they have lower levels of drug intake in operant tasks when directly compared to Lewis [57–60], and as compared to reports with Sprague-Dawley rats, including those from our laboratory [29,30,61]. We observed low levels of responding in the current study by non-Tg rats even with a FR1 schedule of reinforcement for methamphetamine self-administration. This response profile was not significantly altered in the Tg rats, for both genotypes exhibited similar rates of task acquisition and stable operant behavior. These outcomes are similar to our previous study with self-administered cocaine [31] in which drug intake did not differ between Tg and non-Tg rats. In contrast, it was recently reported that Tg rats were more sensitive to lower doses of cocaine (but not heroin) than non-Tg rats, after stable self-administration behavior had been acquired [62]. Several protocol differences may explain these divergent outcomes, e.g., McIntosh *et al*. [62] used different drug doses for each genotype during acquisition of self-administration, whereas we used the same dose of methamphetamine for both genotypes. The contribution of HIV-1 proteins to reward-motivated behavior is complex, e.g., overexpression of the HIV-1 protein Tat potentiates ethanol and cocaine-induced conditioned place preference in mice [63,64]. Thus, additional studies in this exciting new field will be exceptionally informative.

HIV-infection in humans results in marked gut pathology [7], but the status of the intestine in methamphetamine abuse is largely unstudied. Here, we reveal a morphological disorganization of the colon tight junction proteins claudin-1 and ZO-1 in methamphetamine self-administering non-Tg rats relative to saline-yoked non-Tg rats. Functional permeability of the colon was not significantly altered by methamphetamine within either non-Tg or Tg groups of rats. These findings indicate that while the exposure levels of methamphetamine employed in the current study is sufficient to alter colonic tight junction protein expression and localization, these effects may be subthreshold to, or compensated for, putative effects on functional permeability. It is possible that barrier function would be compromised with higher doses of methamphetamine or longer methamphetamine exposure. The moderate levels of methamphetamine intake by the rats used in the current study may have contributed to the absence of an observable interaction of HIV-1 proteins and methamphetamine. Alternatively, the effect of the HIV-1 proteins alone may have been sufficiently robust, such that a ‘ceiling’ effect reflects the lack of enhancement by methamphetamine in the current comorbid model. Regardless, there is literature showing that HIV-1 proteins and methamphetamine may have both distinct and common mechanisms for disrupting tight junction protein expression and/or production. HIV-1 proteins stimulate the production of the pro-inflammatory cytokine TNF-α by epithelial cells, which can activate NF-κB followed by marked reduction in the tight junction protein expression that is due to reduced transcription [65,66]. HIV-1 proteins also stimulate matrix metalloproteinase (MMP) and proteasome activity, which can increase the degradation of barrier proteins that impact barrier continuity [67,68]. Methamphetamine has pro-inflammatory effects in brain [69] and detrimental effects on blood-brain barrier integrity *via* activation of MMP-9 in rodents [70,71]. It is plausible that the capacity of methamphetamine to alter intestinal tight junctions may also involve the pro-inflammatory cascades or increased MMP pathways. Future studies using intestinal epithelial cell lines with HIV-1 proteins and physiologically relevant doses of methamphetamine would be useful in identifying the mechanistic pathways for proposed barrier disruption.

The observation that the colon is a pathological site for HIV-1 proteins and methamphetamine has translational relevance in several domains. First, indices of gastrointestinal pathology may aid in the early diagnosis of brain pathologies. For example, in Parkinson’s disease, gastrointestinal dysfunction precedes motor symptoms [72], and enteric α-synuclein status may serve as early markers of this disease [73]. We have preliminary findings showing that enteric α-synuclein is increased in methamphetamine self-administering Sprague-Dawley rats [74]. Moreover, the preclinical outcomes observed in the current study support case reports indicating that gut pathology is a consequence of methamphetamine abuse [20–23], and illustrate that this field of study would be a fruitful pursuit toward understanding the exaggerated pathology that is associated with comorbid methamphetamine use and HIV-infection [75–77]. Finally, our findings support the utility of HIV-1 Tg rat models for studies on gut dysfunction related to HIV-infected individuals on cART and the consequences of this infection in the context of methamphetamine abuse.

## References

[1] Knox TA, Spiegelman D, Skinner SC, Gorbach S (2000) Diarrhea and abnormalities of gastrointestinal function in a cohort of men and women with HIV infection. Am J Gastroenterol 95: 3482–3489. S0002-9270(00)02158-4 [pii]; doi: 10.1111/j.1572-0241.2000.03365.x 11151881 10.1111/j.1572-0241.2000.03365.x

[2] Bhaijee F, Subramony C, Tang SJ, Pepper DJ (2011) Human immunodeficiency virus-associated gastrointestinal disease: common endoscopic biopsy diagnoses. Patholog Res Int 2011: 247923 doi: 10.4061/2011/247923 21559197 10.4061/2011/247923 PMC3090068

[3] Kotler DP, Gaetz HP, Lange M, Klein EB, Holt PR (1984) Enteropathy associated with the acquired immunodeficiency syndrome. Ann Intern Med 101: 421–428. 6476631 10.7326/0003-4819-101-4-421

[4] Brenchley JM, Douek DC (2008) HIV infection and the gastrointestinal immune system. Mucosal Immunol 1: 23–30. mi20071 [pii]; doi: 10.1038/mi.2007.1 19079157 10.1038/mi.2007.1 PMC2777614

[5] Chun TW, Carruth L, Finzi D, Shen X, DiGiuseppe JA, Taylor H, etl al (1997) Quantification of latent tissue reservoirs and total body viral load in HIV-1 infection. Nature 387: 183–188. doi: 10.1038/387183a0 9144289 10.1038/387183a0

[6] Finzi D, Blankson J, Siliciano JD, Margolick JB, Chadwick K, Pierson T, et al (1999) Latent infection of CD4+ T cells provides a mechanism for lifelong persistence of HIV-1, even in patients on effective combination therapy. Nat Med 5: 512–517. doi: 10.1038/8394 10229227 10.1038/8394

[7] Mudd JC, Brenchley JM (2016) Gut mucosal barrier dysfunction, microbial dysbiosis, and their role in HIV-1 disease progression. J Infect Dis 214 Suppl 2: S58–S66. jiw258 [pii]; doi: 10.1093/infdis/jiw258 27625432 10.1093/infdis/jiw258 PMC5021240

[8] Anderson JM, Van Itallie CM (2009) Physiology and function of the tight junction. Cold Spring Harb Perspect Biol 1: a002584 doi: 10.1101/cshperspect.a002584 20066090 10.1101/cshperspect.a002584 PMC2742087

[9] Lu Z, Ding L, Lu Q, Chen YH (2013) Claudins in intestines: Distribution and functional significance in health and diseases. Tissue Barriers 1: e24978 doi: 10.4161/tisb.24978 2013TISSBARRIER011R [pii]. 24478939 10.4161/tisb.24978 PMC3879173

[10] Reinhold AK, Rittner HL (2017) Barrier function in the peripheral and central nervous system-a review. Pflugers Arch 469: 123–134. doi: 10.1007/s00424-016-1920-8 27957611 10.1007/s00424-016-1920-8

[11] Estes JD, Harris LD, Klatt NR, Tabb B, Pittaluga S, Paiardini M, et al (2010) Damaged intestinal epithelial integrity linked to microbial translocation in pathogenic simian immunodeficiency virus infections. PLoS Pathog 6: e1001052 doi: 10.1371/journal.ppat.1001052 20808901 10.1371/journal.ppat.1001052 PMC2924359

[12] Tincati C, Douek DC, Marchetti G (2016) Gut barrier structure, mucosal immunity and intestinal microbiota in the pathogenesis and treatment of HIV infection. AIDS Res Ther 13: 19 doi: 10.1186/s12981-016-0103-1 103 [pii]. 27073405 10.1186/s12981-016-0103-1 PMC4828806

[13] Passaro RC, Pandhare J, Qian HZ, Dash C (2015) The complex interaction between methamphetamine abuse and HIV-1 pathogenesis. J Neuroimmune Pharmacol 10: 477–486. doi: 10.1007/s11481-015-9604-2 25850893 10.1007/s11481-015-9604-2 PMC4779551

[14] Bowyer JF, Ali S (2006) High doses of methamphetamine that cause disruption of the blood-brain barrier in limbic regions produce extensive neuronal degeneration in mouse hippocampus. Synapse 60: 521–532. doi: 10.1002/syn.20324 16952162 10.1002/syn.20324

[15] Kiyatkin EA, Sharma HS (2009) Acute methamphetamine intoxication: brain hyperthermia, blood-brain barrier, brain edema, and morphological cell abnormalities. Int Rev Neurobiol 88: 65–100. S0074-7742(09)88004-5 [pii]; doi: 10.1016/S0074-7742(09)88004-5 19897075 10.1016/S0074-7742(09)88004-5 PMC3145326

[16] Kousik SM, Graves SM, Napier TC, Zhao C, Carvey PM (2011) Methamphetamine-induced vascular changes lead to striatal hypoxia and dopamine reduction. Neuroreport 22: 923–928. doi: 10.1097/WNR.0b013e32834d0bc8 21979424 10.1097/WNR.0b013e32834d0bc8 PMC3208791

[17] Martins T, Baptista S, Goncalves J, Leal E, Milhazes N, Borges F, et al (2011) Methamphetamine transiently increases the blood-brain barrier permeability in the hippocampus: role of tight junction proteins and matrix metalloproteinase-9. Brain Res 1411: 28–40. S0006-8993(11)01271-6 [pii]; doi: 10.1016/j.brainres.2011.07.013 21803344 10.1016/j.brainres.2011.07.013

[18] Northrop NA, Yamamoto BK (2015) Methamphetamine effects on blood-brain barrier structure and function. Front Neurosci 9: 69 doi: 10.3389/fnins.2015.00069 25788874 10.3389/fnins.2015.00069 PMC4349189

[19] Turowski P, Kenny BA (2015) The blood-brain barrier and methamphetamine: open sesame? Front Neurosci 9: 156 doi: 10.3389/fnins.2015.00156 25999807 10.3389/fnins.2015.00156 PMC4419855

[20] Beyer KL, Bickel JT, Butt JH (1991) Ischemic colitis associated with dextroamphetamine use. J Clin Gastroenterol 13: 198–201. 2033228 10.1097/00004836-199104000-00016

[21] Dirkx CA, Gerscovich EO (1998) Sonographic findings in methamphetamine-induced ischemic colitis. J Clin Ultrasound 26: 479–482. doi: 10.1002/(SICI)1097-0096(199811/12)26:9<479::AID-JCU9>3.0.CO;2-K [pii]. 9800164 10.1002/(sici)1097-0096(199811/12)26:9<479::aid-jcu9>3.0.co;2-k

[22] Holubar SD, Hassinger JP, Dozois EJ, Masuoka HC (2009) Methamphetamine colitis: a rare case of ischemic colitis in a young patient. Arch Surg 144: 780–782. 144/8/780 [pii]; doi: 10.1001/archsurg.2009.139 19687384 10.1001/archsurg.2009.139

[23] Link DP, Chi YW (2011) Massive hematochezia: a complication of methamphetamine-induced vasculitis treated by transcatheter hemostasis. Case Rep Radiol 2011: 919236 doi: 10.1155/2011/919236 22606562 10.1155/2011/919236 PMC3350210

[24] Peng J, Vigorito M, Liu X, Zhou D, Wu X, Chang SL (2010) The HIV-1 transgenic rat as a model for HIV-1 infected individuals on HAART. J Neuroimmunol 218: 94–101. S0165-5728(09)00382-8 [pii]; doi: 10.1016/j.jneuroim.2009.09.014 19913921 10.1016/j.jneuroim.2009.09.014

[25] Field KJ, White WJ, Lang CM (1993) Anaesthetic effects of chloral hydrate, pentobarbitone and urethane in adult male rats. Lab Anim 27: 258–269. doi: 10.1258/002367793780745471 8366672 10.1258/002367793780745471

[26] Huske C, Sander SE, Hamann M, Kershaw O, Richter F, Richter A (2016) Towards optimized anesthesia protocols for stereotactic surgery in rats: Analgesic, stress and general health effects of injectable anesthetics. A comparison of a recommended complete reversal anesthesia with traditional chloral hydrate monoanesthesia. Brain Res 1642: 364–375. S0006-8993(16)30214-1 [pii]; doi: 10.1016/j.brainres.2016.04.019 27067188 10.1016/j.brainres.2016.04.019

[27] Whelan G, Flecknell PA (1992) The assessment of depth of anaesthesia in animals and man. Lab Anim 26: 153–162. doi: 10.1258/002367792780740602 1501428 10.1258/002367792780740602

[28] Ghita AM, Parvu D, Sava R, Georgescu L, Zagrean L (2013) Analysis of the visual evoked potential in anesthesia with sevoflurane and chloral hydrate: (Variability of amplitudes, latencies and morphology of VEP with the depth of anesthesia). J Med Life 6: 214–225. 23904886 PMC3725452

[29] Graves SM, Napier TC (2011) Mirtazapine alters cue-associated methamphetamine seeking in rats. Biol Psychiatry 69: 275–281. S0006-3223(10)01001-2 [pii]; doi: 10.1016/j.biopsych.2010.09.032 21093851 10.1016/j.biopsych.2010.09.032 PMC3015001

[30] Graves SM, Napier TC (2012) SB 206553, a putative 5-HT2C inverse agonist, attenuates methamphetamine-seeking in rats. BMC Neurosci 13: 65 1471-2202-13-65 [pii]; doi: 10.1186/1471-2202-13-65 22697313 10.1186/1471-2202-13-65 PMC3441362

[31] Wayman WN, Chen L, Hu XT, Napier TC (2016) HIV-1 transgenic rat prefrontal cortex hyper-excitability is enhanced by cocaine self-administration. Neuropsychopharmacology 41: 1965–1973. npp2015366 [pii]; doi: 10.1038/npp.2015.366 26677947 10.1038/npp.2015.366 PMC4908633

[32] Keshavarzian A, Farhadi A, Forsyth CB, Rangan J, Jakate S, Shaikh M, et al (2009) Evidence that chronic alcohol exposure promotes intestinal oxidative stress, intestinal hyperpermeability and endotoxemia prior to development of alcoholic steatohepatitis in rats. J Hepatol 50: 538–547. S0168-8278(08)00797-6 [pii]; doi: 10.1016/j.jhep.2008.10.028 19155080 10.1016/j.jhep.2008.10.028 PMC2680133

[33] Forsyth CB, Farhadi A, Jakate SM, Tang Y, Shaikh M, Keshavarzian A (2009) Lactobacillus GG treatment ameliorates alcohol-induced intestinal oxidative stress, gut leakiness, and liver injury in a rat model of alcoholic steatohepatitis. Alcohol 43: 163–172. S0741-8329(09)00012-3 [pii]; doi: 10.1016/j.alcohol.2008.12.009 19251117 10.1016/j.alcohol.2008.12.009 PMC2675276

[34] Farhadi A, Keshavarzian A, Fields JZ, Sheikh M, Banan A (2006) Resolution of common dietary sugars from probe sugars for test of intestinal permeability using capillary column gas chromatography. J Chromatogr B Analyt Technol Biomed Life Sci 836: 63–68. S1570-0232(06)00254-6 [pii]; doi: 10.1016/j.jchromb.2006.03.046 16621740 10.1016/j.jchromb.2006.03.046

[35] Shaikh M, Rajan K, Forsyth CB, Voigt RM, Keshavarzian A (2015) Simultaneous gas-chromatographic urinary measurement of sugar probes to assess intestinal permeability: use of time course analysis to optimize its use to assess regional gut permeability. Clin Chim Acta 442: 24–32. S0009-8981(15)00006-6 [pii]; doi: 10.1016/j.cca.2014.12.040 25591964 10.1016/j.cca.2014.12.040 PMC4339548

[36] Bradford MM (1976) A rapid and sensitive method for the quantification of microgram quantities of protein utilizing the principle of protein-dye binding. Anal Biochem 72: 248–254. 942051 10.1016/0003-2697(76)90527-3

[37] Persons AL, Dodiya HB, Forsyth CB, Keshavarzian A, Napier TC (2014) Assessment of the gut-mucosal barrier in adult HIV-1 transgenic rats. J Neuroimmune Pharmacol 9: 44.

[38] Chung CY, Alden SL, Funderburg NT, Fu P, Levine AD (2014) Progressive proximal-to-distal reduction in expression of the tight junction complex in colonic epithelium of virally-suppressed HIV+ individuals. PLoS Pathog 10: e1004198 doi: 10.1371/journal.ppat.1004198 PPATHOGENS-D-13-03109 [pii]. 24968145 10.1371/journal.ppat.1004198 PMC4072797

[39] Tincati C, Merlini E, Braidotti P, Ancona G, Savi F, Tosi D, et al (2016) Impaired gut junctional complexes feature late-treated individuals with suboptimal CD4+ T-cell recovery upon virologically suppressive combination antiretroviral therapy. AIDS 30: 991–1003. doi: 10.1097/QAD.0000000000001015 00002030-201604240-00002 [pii]. 27028142 10.1097/QAD.0000000000001015

[40] Denton PW, Garcia JV (2011) Humanized mouse models of HIV infection. AIDS Rev 13: 135–148. 21799532 PMC3741405

[41] Zhang YG, Wu S, Lu R, Richards MH, Huelsmann EJ, Lacek AT, et al (2015) HIV Infection leads to redistribution of leaky claudin-2 in the intestine of humanized SCID IL-2R(-/-) Hu-PBMC Mice. AIDS Res Hum Retroviruses 31: 774–775. doi: 10.1089/aid.2014.0341 25853489 10.1089/aid.2014.0341

[42] Brehm MA, Shultz LD, Luban J, Greiner DL (2013) Overcoming current limitations in humanized mouse research. J Infect Dis 208 Suppl 2: S125–S130. jit319 [pii]; doi: 10.1093/infdis/jit319 24151318 10.1093/infdis/jit319 PMC3807974

[43] Reid W, Sadowska M, Denaro F, Rao S, Foulke J Jr., Hayes N, et al (2001) An HIV-1 transgenic rat that develops HIV-related pathology and immunologic dysfunction. Proc Natl Acad Sci U S A 98: 9271–9276. doi: 10.1073/pnas.161290298 98/16/9271 [pii]. 11481487 10.1073/pnas.161290298 PMC55410

[44] Vigorito M, Connaghan KP, Chang SL (2015) The HIV-1 transgenic rat model of neuroHIV. Brain Behav Immun 48: 336–349. S0889-1591(15)00062-8 [pii]; doi: 10.1016/j.bbi.2015.02.020 25733103 10.1016/j.bbi.2015.02.020 PMC4753047

[45] Banerjee A, Abdelmegeed MA, Jang S, Song BJ (2015) Increased sensitivity to binge alcohol-induced gut leakiness and inflammatory liver disease in HIV transgenic rats. PLoS ONE 10: e0140498 doi: 10.1371/journal.pone.0140498 PONE-D-15-27745 [pii]. 26484872 10.1371/journal.pone.0140498 PMC4618849

[46] Honda J, Iijima K, Asanuma K, Ara N, Shiroki T, Kondo Y, et al (2016) Estrogen enhances esophageal barrier function by potentiating occludin expression. Dig Dis Sci 61: 1028–1038. doi: 10.1007/s10620-015-3980-6 26660903 10.1007/s10620-015-3980-6

[47] Wallace DR, Dodson S, Nath A, Booze RM (2006) Estrogen attenuates gp120- and tat1-72-induced oxidative stress and prevents loss of dopamine transporter function. Synapse 59: 51–60. doi: 10.1002/syn.20214 16237680 10.1002/syn.20214

[48] Arrieta MC, Bistritz L, Meddings JB (2006) Alterations in intestinal permeability. Gut 55: 1512–1520. 55/10/1512 [pii]; doi: 10.1136/gut.2005.085373 16966705 10.1136/gut.2005.085373 PMC1856434

[49] Patel S, Leibrand CR, Palasuberniam P, Couraud PO, Weksler B, Jahr FM, et al (2017) Effects of HIV-1 Tat and methamphetamine on blood-brain barrier integrity and function in vitro. Antimicrob Agents Chemother. AAC.01307-17 [pii]; doi: 10.1128/AAC.01307-17 28893794 10.1128/AAC.01307-17 PMC5700307

[50] Liu X, Chang L, Vigorito M, Kass M, Li H, Chang SL (2009) Methamphetamine-induced behavioral sensitization is enhanced in the HIV-1 transgenic rat. J Neuroimmune Pharmacol 4: 309–316. doi: 10.1007/s11481-009-9160-8 19444617 10.1007/s11481-009-9160-8

[51] Kass MD, Liu X, Vigorito M, Chang L, Chang SL (2010) Methamphetamine-induced behavioral and physiological effects in adolescent and adult HIV-1 transgenic rats. J Neuroimmune Pharmacol 5: 566–573. doi: 10.1007/s11481-010-9221-z 20532992 10.1007/s11481-010-9221-z PMC4899043

[52] Moran LM, Aksenov MY, Booze RM, Webb KM, Mactutus CF (2012) Adolescent HIV-1 transgenic rats: evidence for dopaminergic alterations in behavior and neurochemistry revealed by methamphetamine challenge. Curr HIV Res 10: 415–424. CHIVR-EPUB-20120511-3 [pii]. 22591365 10.2174/157016212802138788 PMC3710450

[53] Pang X, Panee J, Liu X, Berry MJ, Chang SL, Chang L (2013) Regional variations of antioxidant capacity and oxidative stress responses in HIV-1 transgenic rats with and without methamphetamine administration. J Neuroimmune Pharmacol 8: 691–704. doi: 10.1007/s11481-013-9454-8 23546885 10.1007/s11481-013-9454-8 PMC3773562

[54] Lominac KD, Sacramento AD, Szumlinski KK, Kippin TE (2012) Distinct neurochemical adaptations within the nucleus accumbens produced by a history of self-administered vs non-contingently administered intravenous methamphetamine. Neuropsychopharmacology 37: 707–722. npp2011248 [pii]; doi: 10.1038/npp.2011.248 22030712 10.1038/npp.2011.248 PMC3260984

[55] Stefanski R, Ladenheim B, Lee SH, Cadet JL, Goldberg SR (1999) Neuroadaptations in the dopaminergic system after active self-administration but not after passive administration of methamphetamine. Eur J Pharmacol 371: 123–135. 10357249 10.1016/s0014-2999(99)00094-1

[56] Jacobs EH, Smit AB, De Vries TJ, Schoffelmeer AN (2003) Neuroadaptive effects of active versus passive drug administration in addiction research. Trends Pharmacol Sci 24: 566–573. S0165-6147(03)00289-X [pii]; doi: 10.1016/j.tips.2003.09.006 14607079 10.1016/j.tips.2003.09.006

[57] Kosten TA, Miserendino MJD, Haile CN, DeCaprio JL, Jatlow PI, Nestler EJ (1997) Acquisition and maintenance of intravenous cocaine self- administration in Lewis and Fischer inbred rat strains. Brain Research 778: 418–429. 9459563 10.1016/s0006-8993(97)01205-5

[58] Kruzich PJ, Xi J (2006) Differences in extinction responding and reinstatement of methamphetamine-seeking behavior between Fischer 344 and Lewis rats. Pharmacol Biochem Behav 83: 391–395. doi: 10.1016/j.pbb.2006.02.021 16574207 10.1016/j.pbb.2006.02.021

[59] Picetti R, Ho A, Butelman ER, Kreek MJ (2010) Dose preference and dose escalation in extended-access cocaine self-administration in Fischer and Lewis rats. Psychopharmacology (Berl) 211: 313–323. doi: 10.1007/s00213-010-1899-3 20559822 10.1007/s00213-010-1899-3 PMC2926930

[60] Picetti R, Caccavo JA, Ho A, Kreek MJ (2012) Dose escalation and dose preference in extended-access heroin self-administration in Lewis and Fischer rats. Psychopharmacology (Berl) 220: 163–172. doi: 10.1007/s00213-011-2464-4 21894484 10.1007/s00213-011-2464-4 PMC3359091

[61] Kousik SM, Carvey PM, Napier TC (2014) Methamphetamine self-administration results in persistent dopaminergic pathology: implications for Parkinson's disease risk and reward-seeking. Eur J Neurosci 40: 2707–2714. doi: 10.1111/ejn.12628 24890790 10.1111/ejn.12628

[62] McIntosh S, Sexton T, Pattison LP, Childers SR, Hemby SE (2015) Increased sensitivity to cocaine self-administration in HIV-1 transgenic rats is associated with changes in striatal dopamine transporter binding. J Neuroimmune Pharmacol 10: 493–505. doi: 10.1007/s11481-015-9594-0 25749646 10.1007/s11481-015-9594-0 PMC4701048

[63] McLaughlin JP, Ganno ML, Eans SO, Mizrachi E, Paris JJ (2014) HIV-1 Tat protein exposure potentiates ethanol reward and reinstates extinguished ethanol-conditioned place preference. Curr HIV Res 12: 415–423. CHRE-EPUB-65842 [pii]. 25760047 10.2174/1570162x1206150311160133

[64] Paris JJ, Carey AN, Shay CF, Gomes SM, He JJ, McLaughlin JP (2014) Effects of Conditional Central Expression of HIV-1 Tat Protein to Potentiate Cocaine-Mediated Psychostimulation and Reward Among Male Mice. Neuropsychopharmacology 39: 380–388. npp2013201 [pii]; doi: 10.1038/npp.2013.201 23945478 10.1038/npp.2013.201 PMC3870789

[65] Nazli A, Chan O, Dobson-Belaire WN, Ouellet M, Tremblay MJ, Gray-Owen SD, et al (2010) Exposure to HIV-1 directly impairs mucosal epithelial barrier integrity allowing microbial translocation. PLoS Pathog 6: e1000852 doi: 10.1371/journal.ppat.1000852 20386714 10.1371/journal.ppat.1000852 PMC2851733

[66] Ma TY, Iwamoto GK, Hoa NT, Akotia V, Pedram A, Boivin MA, et al (2004) TNF-alpha-induced increase in intestinal epithelial tight junction permeability requires NF-kappa B activation. Am J Physiol Gastrointest Liver Physiol 286: G367–G376. doi: 10.1152/ajpgi.00173.2003 286/3/G367 [pii]. 14766535 10.1152/ajpgi.00173.2003

[67] Vermeer PD, Denker J, Estin M, Moninger TO, Keshavjee S, Karp P, et al (2009) MMP9 modulates tight junction integrity and cell viability in human airway epithelia. Am J Physiol Lung Cell Mol Physiol 296: L751–L762. 90578.2008 [pii]; doi: 10.1152/ajplung.90578.2008 19270179 10.1152/ajplung.90578.2008 PMC2681350

[68] Lischper M, Beuck S, Thanabalasundaram G, Pieper C, Galla HJ (2010) Metalloproteinase mediated occludin cleavage in the cerebral microcapillary endothelium under pathological conditions. Brain Res 1326: 114–127. S0006-8993(10)00432-4 [pii]; doi: 10.1016/j.brainres.2010.02.054 20197061 10.1016/j.brainres.2010.02.054

[69] Goncalves J, Martins T, Ferreira R, Milhazes N, Borges F, Ribeiro CF, et al (2008) Methamphetamine-induced early increase of IL-6 and TNF-alpha mRNA expression in the mouse brain. Ann N Y Acad Sci 1139: 103–111. NYAS1139043 [pii]; doi: 10.1196/annals.1432.043 18991854 10.1196/annals.1432.043

[70] Northrop NA, Halpin LE, Yamamoto BK (2016) Peripheral ammonia and blood brain barrier structure and function after methamphetamine. Neuropharmacology 107: 18–26. S0028-3908(16)30090-9 [pii]; doi: 10.1016/j.neuropharm.2016.03.018 26972828 10.1016/j.neuropharm.2016.03.018 PMC5264515

[71] Goncalves J, Leitao RA, Higuera-Matas A, Assis MA, Coria SM, Fontes-Ribeiro C, et al (2017) Extended-access methamphetamine self-administration elicits neuroinflammatory response along with blood-brain barrier breakdown. Brain Behav Immun 62: 306–317. S0889-1591(17)30058-2 [pii]; doi: 10.1016/j.bbi.2017.02.017 28237710 10.1016/j.bbi.2017.02.017

[72] Sung HY, Park JW, Kim JS (2014) The frequency and severity of gastrointestinal symptoms in patients with early Parkinson's disease. J Mov Disord 7: 7–12. doi: 10.14802/jmd.14002 jmd-7-1-7-2 [pii]. 24926404 10.14802/jmd.14002 PMC4051727

[73] Mukherjee A, Biswas A, Das SK (2016) Gut dysfunction in Parkinson's disease. World J Gastroenterol 22: 5742–5752. doi: 10.3748/wjg.v22.i25.5742 27433087 10.3748/wjg.v22.i25.5742 PMC4932209

[74] Napier TC, Kousik SM, Kelly LP, Graves SM, Persons AL (2013) Rats that self-administer methamphetamine show Parkinson's disease-like inflammation and alpha-synuclein pathology in the brain and colon. Program No 240 12 Neuroscience Meeting Planner San Diego, CA Society for Neuroscience Online.

[75] Ferris MJ, Mactutus CF, Booze RM (2008) Neurotoxic profiles of HIV, psychostimulant drugs of abuse, and their concerted effect on the brain: Current status of dopamine system vulnerability in NeuroAIDS. Neurosci Biobehav Rev 32: 883–909. doi: 10.1016/j.neubiorev.2008.01.004 18430470 10.1016/j.neubiorev.2008.01.004 PMC2527205

[76] Nath A (2010) Human immunodeficiency virus-associated neurocognitive disorder: pathophysiology in relation to drug addiction. Ann N Y Acad Sci 1187: 122–128. NYAS5277 [pii]; doi: 10.1111/j.1749-6632.2009.05277.x 20201849 10.1111/j.1749-6632.2009.05277.x

[77] Buch S, Yao H, Guo M, Mori T, Su TP, Wang J (2011) Cocaine and HIV-1 interplay: molecular mechanisms of action and addiction. J Neuroimmune Pharmacol. doi: 10.1007/s11481-011-9297-0 21766222 10.1007/s11481-011-9297-0 PMC3208732

